# Report of the Medical Section of the British Association for the Advancement of Science. Held at Bristol, in August, 1836

**Published:** 1836-10

**Authors:** 


MEETING OF THE BRITISH ASSOCIATION FOR THE ADVANCEMENT OF SCIENCE.
*Held at Bristol, in August,* 1836.

MEDICAL SECTION.

*President,* Dr. Roget:
*Vice Presidents,* Dr. Bright, Dr. Macartney;
*Secretaries,* Dr. Symonds, G. D. Fripp, Esq.

ABSTRACT OF THE PROCEEDINGS OF THE MEDICAL SECTION.

THE proceedings of the Section were opened on Monday, the 22d of August, by an address from its President, Dr. Roget, on the general objects of the Association, and those of the Medical Section in particular. Those objects he stated to be the collection and concentration of information, and its subsequent diffusion, after having been carefully sifted and having received the stamp of the Association.

1. The Report of the Dublin Committee appointed to investigate the Pathology of the Brain and Nervous System was produced by Dr. O'Beirne, who stated that the present must be considered only as a provisional Report, as the committee had been chiefly occupied with the collection of numerous cases of injuries of the brain, and lesions of individual nerves, the particulars of which cases had been carefully registered; and they are anxious to procure a still greater number before they venture to form any general inferences.

2. Dr. O'Beirne then communicated an abstract of an unpublished work on Tetanus. He commenced with an account of the opportunities which he had enjoyed of observing this disease, which were numerous and extensive, and of the circumstances which induced him to commence and prosecute the enquiry. Dr. O'Beirne repudiates all specific divisions of the disease, except that which distinguishes the *traumatic* (or symptomatic) from the *atraumatic* (or idiopathic). He allows of no such varieties as *Trismus* and *Pleurosthotonos*, recognising only *Opisthotonos*, which is the more common form, and *Emprosthotonos*, which he agrees with authors in considering very rare. He proposed to arrange particular specimens of the disease as being *peracute*, *acute*, *subacute*, and *chronic*; and then went into a consideration of the causes, respecting which his views were in accordance with most authorities.

The extreme periods of accession of the traumatic species were, according to his experience, the fourth day and the seventeenth; he stated that it never occurred after the cicatrization had been completed, or during the existence of active inflammation in the wound, and that it never succeeded a wound which did not penetrate below the skin, to the muscles or fascia. The general character of the disease he finds to be the same in all climates, countries, and ages; but the atraumatic species supervenes much earlier than the traumatic.

He has no confidence in any reputed premonitory symptoms, and finds that in no instance is it ushered in or attended by symptoms peculiar to febrile diseases or eruptions on the skin, except quickness of pulse, which in the atraumatic species is permanent. He denies the existence of any tendency to crises or sudden disappearance, and states that recovery takes place slowly, the period varying from eighteen days to seven or eight weeks.

The only true pathognomonic sign is a *peculiar expression of the countenance*, long retained after the attack, and even after death, and not indicative of any particular mental emotion. An engraved representation of the face of a patient during and between the tetanic paroxysms was exhibited.

The symptoms of the disease in both species were minutely stated, and arranged under three stages. The only constant morbid appearances are distention of the cæcum and colon, and rigid contraction of the rectum. In the emprosthotonic variety, an extremely contracted state of the stomach is occasionally noticed.

Dr. O'Beirne lays stress upon the amount of pain in opisthotonos being much less than is generally supposed, and declares the symptoms and course to be more uniform in this than in any other malady. In his observations on the diagnosis, he remarked upon the extraordinary extent to which this disease had been confounded with others, and enumerated the spurious cases under several heads. He particularly mentioned that, if he were to find in a patient the angles of the mouth drawn either upwards or downwards, he should conclude that the case was not a true specimen of the disease.

With respect to the seat of tetanus, Dr. O'B. expressed his belief that the anterior column of the spinal cord, and probably the thalami optici and corpora striata, are the parts in the affections of which we must look for the pathology of the disease; but he considers that, in emprosthotonos, the pneumogastric nerve is also affected. The change in these parts is functional only, and not connected with inflammation or organic lesion. All the symptoms are therefore the product of muscular action, dependent upon an accumulation or increased intensity of the *motific principle*, residing in the anterior spinal column.

The remedial measure on which Dr. O'B. places most reliance is the use of tobacco enemata, thrown into the colon by means of a flexible tube introduced through the rectum. This method is detailed in his work on Defœcation, which he stated to have been the offspring of his researches upon tetanus. He presented a list of twenty cases, eleven of which recovered; and concluded his paper by adverting to the disease as it occurs in the horse, in the treatment of which, his method, in the hands of veterinary surgeons, had been attended with success.

A discussion then ensued, in which Dr. Yelloly, Dr. Reid, Dr. Wallis, Mr. Bracy Clark, Dr. Fowler, Mr. King, Mr. Hetling, Dr. Symonds, Mr. Greeves, Dr. Howell, Mr. Carmichael, and Mr. Broughton, severally took a part. To us it appeared that the difference between Dr. O'Beirne and those who opposed his views was principally one of words, depending upon the restricted or extended sense in which the term tetanus was respectively employed by them.

3. A case of Aneurism of the Arteria Innominata and Thoracic Aorta, communicated by Sir David Dickson, was read by Dr. Symonds. This paper was accompanied by a drawing representing the diseased parts.

Tuesday.—1. The first paper read was entitled "Observations on Remedies for Diseases of the Brain," by Dr. Prichard.

The principal object of this paper was to recommend the employment of a peculiar mode of counter-irritation in all those forms of cerebral disease which are accompanied by coma, stupor, or diminished sensibility, excluding those which are attended with excitement. This method consists in the formation of a *long issue*, better made by the scalpel than by an escharotic, in the direction of the sagittal suture. The details of a case of complete amaurosis unsuccessfully combated by the antiphlogistic and mercurial treatment, but in which sight began to return immediately upon the establishment of suppuration in an issue of this description, were given; and the patient, to whom his sight has been perfectly restored, was submitted to the examination of the Section.

2. A communication was then read by Dr. Houston, of Dublin, on a Human Fœtus without Heart or Lungs. The case was one of twins, between the seventh and eighth months. The placentæ were intimately united, but the line of their junction was distinct. Each fœtus had its own membranes and umbilical cord, the latter being inserted into the placenta at some distance from each other. The imperfect fœtus was acephalous, as in other instances of the kind, but was alive at the time of birth; there was no subdivision of chest and abdomen; the spinal canal began at the sixth cervical vertebra; there was no heart, lungs, liver, or stomach; the lower intestines contained meconium; the kidneys existed, but only one ureter; and there was general œdema of the whole body. The state of the circulating apparatus had been carefully examined by Dr. H., and appeared to him to lead to some important conclusions. The cord was short, and the umbilical vein, which was nearly straight, communicated with the right hypogastric, which joined the vena cava in the usual manner. From this vessel branches proceeded throughout the system, *all of which were totally devoid of valves.* The arterial system arising from the aorta was in like manner distributed through the body, but had nowhere any communication with the venous system, except by the capillaries; so that, by whatever vessels the blood entered the body, by the same it must have been distributed. A round tumour, which existed in the substance of the cord, outside the umbilicus, had produced effects on the vessels calculated to throw light on the course of the circulation; the vein was varicose from the tumour as far as the placenta, and the arteries were dilated on the side next the body of the fœtus.

Dr. Houston then adverted to the researches of various anatomists on the circulation of the acardiac fœtus, and especially to those of Sir A. Cooper, who maintains, from the examination of a recent case, that the umbilical vessels of two fœtuses anastomose freely in the placenta, and that the circulation in the imperfect embryo is carried on by the action of the heart of its companion in utero. If this view be correct, the heart of the perfect fœtus ought to be nearly double its usual size, or the child itself small,—neither of which is the case; the umbilical artery of the imperfect fœtus would then serve the purpose of the vein in carrying the blood *from* the placenta, which in this case it did not appear to have done; and a large quantity of the blood would be continually circulating without the renovating influence of the placenta. The presence of a second placenta, however imperfect or however connected with the other, is incompatible with the direct transmission of blood from the perfect to the imperfect fœtus, and with the idea that the latter is a mere offset from the former. The effects produced by the tumour on the vessels of the cord, and the absence of valves in the veins throughout the body, were adduced by Dr. Houston as strong evidence in favour of his opinion that the circulation of the *cord* of the monster was regular, but that in its *body* the functions of the arteries and veins had been inverted. He considers that the presence of an anastomosis between the cords should lead rather to the inference that the blood traverses both placentæ in the same course, than that it takes a direction in the one different from that which it follows in the other. Dr. H. suggested several good reasons in support of his opinion that the heart of the perfect fœtus can exert little influence upon the vessels of the other; and expressed his opinion that we must look to some other cause than *vis à tergo* for the accomplishment of this object. The theory of "vital attractions and repulsions," though conveyed in terms which may be considered rather as expressive of the facts than as explanatory of them, probably approaches nearer to the truth than any other which has yet been broached. With respect to the influence which the action of the vessels themselves has upon the circulation, Dr. Houston mentioned a case in which injection passed very freely through the vessels of a mortified limb, which were entirely free from coagulum or other obstruction; but the blood had not passed through them during life, though the action of the heart was unimpaired.

Dr. Prichard and Dr. Carson objected to the inference drawn by Dr. Houston from the latter case. Dr. Macartney stated that he had more than once endeavoured to inject a gangrenous limb immediately after its removal, but had been unsuccessful.

Mr. Carpenter pointed out the very striking analogies which are presented by the circulation in vegetables, all of which favour the view that the motion of fluids in vessels is in a great degree independent of a central organ of impulsion, and is closely connected with the changes which take place in the capillary vessels. He also alluded to the fact that there was no case on record in which an acardiac fœtus existed without a perfect fœtus.

Dr. Houston remarked on the latter point, that he was strongly disposed to believe that the partial union of the placentæ was the cause of the monstrosity.

3. The next paper read was on Tubercle, by Mr. Carmichael, of Dublin.

Mr. Carmichael commenced with some remarks upon the great prevalence of these formations, and then proceeded to detail their appearances according to the descriptions of Laennec and Carswell. He alluded to the use of the term Scrofula, which he considers a cloak for ignorance, and having stated that Drs. Todd, Clark, and Carswell, believe in the identity of Scrofula and Tubercle, disputed this position, and likewise their opinion that tubercles are inorganic deposits. Among other objections, he urged the inconsistency of representing enlarged cervical glands and pulmonary tubercles as identical, since it is well known that the former may be injected, but not the latter;—and of maintaining the non-inflammatory origin of tubercles, together with the view that these bodies are lifeless matter; since, if such is their nature, they must excite inflammation in the tissues which contain them. He allows, however, that the scrofulous constitution disposes to Tubercle, but only in the same manner as to Cancer. Mr. C. next adverted to the generally recognised connexion between Scrofula and disordered digestion, and claimed the priority of this observation, by reference to a work which he published in 1810. He then proceeded to argue at considerable length in favour of the parasitic origin of tubercles, pointed out the absence of vascular communication between these bodies and surrounding parts, and observed that so long as the former retain their vitality, no inflammation takes place.

The author declared his opinion that Carcinoma must likewise be arranged among the Entozoa, and having indicated the division of a cancerous formation into a medullary and cartilaginous portion,—assigned to the former an independent vitality, the latter being only a barrier which nature sets up against the parasite,—and shewed that the containing cyst belongs to the surrounding tissue. The cartilaginous portion, he stated, might be injected, but not the medullary. Tubercles he considers more allied to Carcinoma than to Scrofula. Having spoken of a difference between Fungus Medullaris and Fungus Hematodes, he proposed to arrange the formations which had passed under review as constituting four species of Entozoa. 1. Tubercles found in the lungs. 2. Tubercles found in the abdominal organs. 3. Fungus Medullaris and Hæmatodes. 4. Cancer.

Mr. Carmichael next considered the exciting causes of Tubercles, and concluded his paper by urging that practitioners must direct their attention rather to the prevention than to the cure of the disease; but allowed that improvement in the general habit may possibly, at the commencement of the disease, tend to its removal.

Dr. Macartney stated several reasons for his dissent from Mr. Carmichael's doctrine of the independent vitality of Tubercle. He considers that the vesicular appearance is confined to the lungs, and is owing to the deposit taking place around the bronchial cells. He also contended, that although tubercular matter itself is not vascular, each deposit is traversed by bands of cellular tissue which may be injected, and by which it is distinguished from a hydated vesicle. He recommended to the attention of anatomists his mode of displaying the vascularity of minute structures, by drying them on glass after having injected them.

Dr. Symonds objected to the manner in which the views of Dr. Carswell, Dr. Clark, and others, had been represented by Mr. Carmichael. They had not appeared to him to maintain the identity of Scrofula and Tubercle, but that they stood in the relation of cause and effect, or of *diathesis* and *disease*. Nor did those authors appear to him to have attributed an identical structure to tubercles in the lungs, and enlarged cervical glands; they had only shewn that tuberculous matter is deposited in the tissue of the glands, and consequently that there is no difficulty in understanding why the latter may be injected, though mere tuberculous substance in the lungs, or elsewhere, cannot. Dr. S. urged, that as the form of tubercles in the lungs depended on the parts in which they were deposited, no argument could be drawn from so accidental a character in favour of the hydatidal doctrine.

Wednesday.—1. Dr. Macartney read the second report of the Dublin Committee appointed to investigate the actions and sounds of the heart. In answer to the questions proposed in the resolutions of the Committee last year, they state that the contraction of the columnæ carneæ occurs at the same precise moment as that of the whole mass of the ventricle, and that the valves are not *tightened* by their contraction, but by the impulse of the blood during systole, although they are drawn towards the auriculo-ventricular axis by the first-mentioned cause. The experiments instituted by them to elucidate the causes of the sounds of the heart, brought them to the same results as have been detailed in the first report; and they suggest that the future investigation of the subject should be prosecuted rather by the observation of disease than by physiological experiments.

2. The report of the London Committee upon the same subject was next read by Dr. Clendinning.

Considering that each sound of the heart might have a single cause, or be the result of a combination of causes, the Committee began their investigations by enumerating all the causes which might be supposed to be concerned; and resolved to vary their experiments so as to exclude each of them in succession.

The following is the result of their researches regarding the causes to which the first sound has been attributed :—1. *Valvular tension.* The action of the auriculo-ventricular valves may be prevented, without the sound being immediately diminished.—2. *Collision of the particles of fluid* amongst themselves and against the sides of the heart. By experiments with a caoutchouc bottle, it was proved that this cannot be one of the causes of the *normal* first sound of the heart; but it appeared probable that it might contribute to the production of the bellows sound, if any obstruction were offered to the motion of the fluid. —3. *Impulse.* This is probably an accessory, but not the principal cause of the first sound.—4. *Muscular tension.* From various experiments it appears that this is the essential cause of the first sound. If a flexible ear tube be applied over the abdominal muscles, the sound of their contraction may be distinctly heard (being probably exaggerated by the cavity beneath), and appears to be of a *systolic* character. A sudden loud sound is heard during the contraction, and continues in a less degree as long as the contraction is kept up.

As to the second sound, the period at which the columnæ carneæ contract, and the cause of the tension of the valves, their conclusions are the same as those of the Dublin Committee.

3. A letter from Dr. Spittal, of Edinburgh, to the Secretary of the Section was read, explaining the cause of no report having been furnished by the Edinburgh Committee.

4. A communication on the Mechanism of the Heart was read by Mr. Greaves, of Nottingham. The author endeavoured to show that the *external* spiral fibres of the ventricles, antagonize the *internal,* and produce active dilatation of the cavity, instead of the passive dilatation which is generally regarded as being produced by the relaxation of the muscular fibres, and the elasticity of the inter-muscular tissue. He referred the mechanical phenomena of the heart's actions to a species of gyratory movement, compensated and regulated by the double spiral curve of the two great arteries, which he regards as acting like the balance spring of a watch.

5. Dr. Roget read some observations on a singular developement of polarizing structure in the crystalline lens after death, communicated by Sir D. Brewster.

The object of the author of this paper was to institute a comparison between the changes produced by age in the polarizing structure of the crystalline lens of animals, with those changes which occur in the same organ after death, when the lens is allowed to indurate by exposure to the air, or is subjected to the continued action of an aqueous fluid, such as distilled water. With this view he examined the optical characters of the coloured luminous rings which appear in consequence of the polarizing properties acquired by the lens under these circumstances. These phenomena he conceives to afford a satisfactory explanation of the changes in the lens which terminate in cataract, a disease which seems to be more prevalent now than in former times. In a letter addressed to Dr. Roget, Sir D. Brewster gave the history of the condition of his own eye, in which he accidentally observed appearances indicating the commencement of the process described in this paper, and detailed the plan which he adopted for counteracting the progress of this affection, by which the organ was restored to its natural and healthy state.

6. Dr. Carson, of Liverpool, then read a paper on Absorption. He arrives at the conclusion that absorption is effected by three sets of vessels ;—that of nutritious matter by the *Lacteals* and *Lymphatics;*—and that of matter which has become useless in the system, by the *Veins.* He imagines that the arteries and veins communicate with each other throughout the system by means of cells, like those of erectile tissue, into which the terminations of each set of vessels open ; and that the nutritious particles deposited by the arteries, displace those which have become useless, and which are taken up by the open mouths of the venous capillaries ; and he believes that veins do not absorb by their parietes. Dr. C. entered into an extensive consideration of the manner in which the contents of the veins were conveyed to the heart, and of the changes which take place in the pulmonary vessels.

Thursday.—1. Dr. Hodgkin read a provisional Report of the Committee for investigating the connexion between Veins and Absorbents. As this report will appear in a complete form in the Transactions of the Association, we think it better not to give any abstract of it at present.

2. A communication on the Functions of Nervous Structure, was read by Dr. Reid, of Dublin. The principal object of this paper was to enforce the method of studying the nervous system under three divisions: viz. the Ganglionic, the Spinal, and the Cerebral systems. Dr. R. pointed out what he conceived to be the principal functions and province of each of these systems, and stated his opinion that all diseases should be arranged, and all remedies selected, according as the latter have their action directed to, and the former are found to affect one or other of these divisions of the nervous system in particular.

3. Mr. Broughton read a report by Dr. Marshall Hall and himself, on the Sensibility of the Glosso-Pharyngeal nerve. They had in a former report arrived at a negative result; viz. that this nerve does not endow the tongue with tactile sensibility or motion, which clearly depend on the fifth and seventh pairs. They now present their experimental researches in proof of the Glosso-Pharyngeal being the true nerve of taste, which completely accord with the observations of Panizza. Some facts in Comparative Anatomy were mentioned corroborative of their views.

4. A communication from Mr. Alcock, on some particulars in the Anatomy of the fifth pair, was read by Dr. Geogheghan.

5. Dr. Macartney presented an account of the organ of voice in the New Holland Ostrich. The peculiar *grunt-like* sound produced by the animal depends, as he has discovered, on the communication of the trachea with a large membranous cell placed under the skin of the neck; a formation peculiar to the animal, and common to both sexes.

6. Dr. Macartney next read a paper on the Pulps of the Teeth, and on Caries of these organs.

7. A communication from Dr. Montgomery, on a newly-discovered Peculiarity in the Structure of the Decidua, was then read by Dr. Geogheghan. The discovery announced in this paper was that of a number of small cuplike elevations on the uterine surface of the decidua uteri, which are found to be little bags, like the suckers of the cuttle-fish in miniature, the bottoms of which are attached to or imbedded in the substance of the decidua; they then bulge out, and terminate by a contracted neck, leaving an open mouth when the membrane is separated from the uterus. They are most numerous at a distance from the placenta, and most evident in the very early period of gestation, before the placenta is formed as a distinct organ. Dr. M. suggests, from having occasionally found them to contain a chylous fluid, whether they may not be receptacles of nutrient matter separated from the maternal blood.

8. A paper on the Functions of the Muscles and Nerves of the Eyeball was read by Mr. Walker. The action of the oblique muscles of the eyeball was explained by the author as rotating the eye inwards, but by opposite rotatory movements; so that, if the eye were rotated in one direction by the action of one of these muscles, it would be returned to its former position by the action of its antagonist; while, if both muscles were in action together, there would be no rotation at all, but a direct drawing of the eye inwards. An explanation of the reason for the complicated muscular apparatus of the eyeball is afforded, in Mr. W.'s opinion, by a reference to the arrangement of the nerves. The distribution of the 3d nerve to the superior, internal, and inferior rectus, and to the inferior oblique, points out the association of these muscles in all corresponding motions of both eyes; and the two other nerves (the 4th and 6th,) with which the two remaining muscles are supplied are required for the direction of the eye outwards, whilst the other is turned inwards, as is the case whenever an object is viewed laterally.

Friday.—1. A communication was made on the Pathological Condition of the Bones in Chronic Rheumatism, by Mr. Adams, of Dublin. The various changes taking place in the extremities of the bones which constitute the joints chiefly attacked by this disease were minutely described, and illustrated by interesting specimens, casts, and drawings.

2. The Report of a Committee appointed in Dublin to pass opinion upon a case exhibited by Mr. Snow Harris to the section at the last meeting of the Association, was read by Dr. Evanson. The committee are decidedly of opinion that this interesting case was not one of fracture of the neck of the femur, as had been supposed, but an instance of the Morbus Coxæ Senilis.

3. Mr. Adams exhibited to the section a very splendid preparation of a case of double Popliteal Aneurism, illustrative of the changes which are produced on the circulation by the ligature of the femoral artery. In this instance the tube of the artery was continuous immediately above and below the situation where the ligature had been applied; and the anastomosing channels were very large. Mr. A. pointed out that the speedy restoration of the tube of the artery accounts for the secondary pulsation which often occurs within two or three days after the operation, until coagulation takes place in the sac. This occurrence has often caused alarm to the surgeon, but unnecessarily, since the cure of aneurism seems to depend rather upon the retardation of the current of blood than upon its total obstruction.

4. Mr. Hetling then exhibited a new instrument for the removal of ligatures from arteries. He showed that, by the use of it when the ligature is applied, the thread may be afterwards cut at any time; and that all the inconveniences arising from the protracted retention of the ligature may be thus avoided.

5. A paper by Mr. Alcock was read, disproving the opinion of some physiologists that the sense of taste is dependent on nerves from the spheno-palatine ganglion, which may be considered a confirmation of the views stated in Mr. Broughton's paper of the preceding day.

6. Dr. R. T. Thomson read a paper on the chemical states of the digestive organs in health and disease. His observations did not appear to us to have led to any novel results.

7. Mr. Gordon exhibited some portions of a very beautiful and correct anatomical model of the human body, carved in ivory, upon which he has been engaged for many years.

8. Dr. Howell communicated a case in which a large portion of the ileum had been passed per anum, the patient surviving more than twelve months; it was illustrated by some interesting drawings.

After a concluding address from Mr. Broughton, (who had taken the chair in the absence of the Presidents and Vice Presidents,) this Section adjourned.

[For the above most valuable and interesting Report we are indebted to two professional friends, who were present during the whole period of the Meeting, and had access to all the documents presented to the Section. We can therefore with confidence present it to our readers, as no less authentic than accurate.—Eds.]

